# A PFAS mixture at environmental relevant concentration induces DNA damage and compromises the in vitro maturation of mouse oocytes

**DOI:** 10.1007/s00204-026-04439-3

**Published:** 2026-05-13

**Authors:** Aleksandra Tatarczuch, Katarzyna Kotarska, Zbigniew Polański, Anna Ptak

**Affiliations:** 1 Laboratory of Physiology and Toxicology of Reproduction, Institute of Zoology and Biomedical Research, Jagiellonian University, Gronostajowa 9, Krakow, 30-387 Poland; 2 Laboratory of Genetics and Evolution, Institute of Zoology and Biomedical Research, Jagiellonian University, Gronostajowa 9, Krakow, 30-387 Poland; 3 Doctoral School of Exact and Natural Sciences, Jagiellonian University, Prof. St. Łojasiewicza 11, Krakow, 30-348 Poland

**Keywords:** Per- and poly-fluoroalkyl substances, Mouse oocyte, Mitochondrial activity, DNA damage, Oxidative balance, *in vitro* maturation

## Abstract

Per- and polyfluoroalkyl substances (PFAS) are persistent environmental pollutants that can accumulate in the human body, including follicular fluid (FF), potentially affecting the function of reproductive cells. We used the mixture of these chemicals at concentrations previously reported in human FF to assess their effects on in vitro maturation, DNA integrity, and mitochondrial function of mouse oocytes. Exposure to PFAS led to meiotic abnormalities, manifested by an increase in the percentage of oocytes arrested at the germinal vesicle breakdown (GVBD) stage and a decrease in the number of oocytes reaching the metaphase II (MII) stage. We demonstrated that arrest at GVBD is associated with Spindle Assembly Checkpoint (SAC) activation as experimental inhibition of SAC restores normal maturation to MII. In addition, we observed increased DNA damage and changes in oxidative balance, including an increase in reduced glutathione (GSH) and a decrease in reactive oxygen species (ROS) levels. However, we found an increase in mitochondrial activity, basal oxygen consumption (OCR), and mitochondrial respiration intensity in immature GV stage oocytes, indicating disturbances in bioenergetic homeostasis. These findings show for the first time that PFAS mixtures, at concentrations relevant to human exposure, interfere with oocyte maturation, genome integrity, and mitochondrial function. The data obtained highlight the need for further research on the toxicity of PFAS mixtures, especially at doses mirroring their environmental presence, and their potential impact on female fertility.

## Introduction

Mammalian female germ cells initiate meiosis during fetal life, but its progression is soon arrested for a prolonged period at the diplotene of the first meiotic division, also known as the germinal vesicle (GV) stage. In adult females, fully grown GV-stage oocytes resume meiosis sequentially in response to hormonal stimulation during each oestrus cycle. Upon resumption of meiosis oocytes enter the final stage of oogenesis known as meiotic maturation. The first morphological sign of this process is the dissolution of the nuclear envelope, known as germinal vesicle breakdown (GVB), followed by chromatin condensation and the formation of the spindle of the first meiotic metaphase (MI). The subsequent anaphase of the first meiotic division is accompanied by asymmetric cytokinesis resulting in the extrusion of the first polar body (PB1). Importantly, under some conditions in which genomic integrity is compromised, the Spindle Assembly Checkpoint (SAC) can arrest progression of meiotic maturation at MI thereby reducing the risk that an oocyte with an abnormal genome completes maturation and subsequently undergoes fertilization (Polański, 2013; Lane and Kauppi 2024). After completion of the first meiotic division oocytes soon progress to the second meiotic metaphase (MII) which marks the end of meiotic maturation. At this stage the cell cycle is arrested again until sperm penetration. During meiotic maturation the oocyte cytoplasm acquires the competence to respond appropriately to sperm entry and thus initiate development. Furthermore, disruption of the progression of this process may result in erroneous chromosome segregation or cause defects in the oocyte genome that can be transmitted to the embryo. Consequently, factors that interfere with meiotic maturation may lead to infertility, miscarriage or birth defects (Wang et al. 2021; Yao et al. 2023).

Per- and polyfluoroalkyl substances (PFAS) are widespread and used in a variety of industrial and consumer products, including cosmetics, pharmaceuticals, and food packaging materials (Ding et al. 2020). These chemicals can cross the blood-follicle barrier and accumulate in the follicular fluid (FF) (Kim et al. 2020) thereby directly affecting the developing oocytes. The PFAS most commonly detected in FF such as perfluorooctane sulfonate (PFOS), perfluorooctanoic acid (PFOA), perfluorohexane sulfonate (PFHxS), perfluorodecanoic acid (PFDA), perfluoroheptanesulfonate (PFHpA), perfluorooctadecanoic acid (PFUnDA) and perfluorononanoic acid (PFNA) have been reported at concentrations ranging from 0.4 ng/ml for PFUnDA to 22.4 ng/ml for PFOS (Kim et al. 2020; Bellavia et al. 2023; Boney et al. 2026). Recent evidence, although limited, suggests that individual PFAS like PFOS, PFOA, and PFHxS may affect oocyte function by inducing oxidative stress and mitochondrial dysfunction thereby impairing oocyte meiotic maturation (Hallberg et al. 2022; Zhang et al. 2022; Feng et al. 2023; Yan et al. 2025). In addition, studies indicate that exposure to PFNA (Jiao et al. 2021) as well as to a mixture of PFOS and PFOA (Yan et al. 2025) leads to DNA damage in oocytes. Epidemiological studies indicate, however, that oocytes are exposed to a more complex composition of these compounds. Therefore, understanding the effects of PFAS mixtures at environmentally relevant concentration on oocyte maturation is of particular importance and warrants further investigation.

The objective of this study was to determine whether a PFAS mixture, with a composition comparable to that detected in human FF (Kim et al. 2020), may affect the function of mouse oocytes at the prophase stage and during in vitro maturation. To this end, we analyzed the progression of the oocytes through meiotic maturation as well as the effect of the PFAS mixture on the induction of oxidative stress, mitochondrial function, and cellular bioenergetics. Particular emphasis was placed on assessing the association between PFAS exposure and DNA damage in oocytes. Such damage could compromise the oocyte genomic integrity, thereby adversely affect embryonic development and potentially contributing to the accumulation of mutations across subsequent generations.

## Materials and methods

### Reagents and treatments

PFOA, PFUnDA, PFNA, PFDA, PFHpA, PFHxS were purchased from Sigma-Aldrich, Co. (St. Louis, MO, USA), whereas PFOS was obtained from Santa Cruz Biotechnology Inc. (Texas, USA). A stock solution of the test compound mixture was prepared by dissolving all components in water for cell culture (WFI; Thermo Fisher Scientific, Inc., USA). This solution was added directly to the culture medium to achieve the following final concentrations (referred to hereafter as Mix) reported in human FF: 224 ng/ml PFOS, 145 ng/ml PFOA, 213 ng/ml PFHxS, 9 ng/ml PFDA, 6 ng/ml PFHpA, 4 ng/ml PFUnDA, and 20 ng/ml PFNA (Kim et al. 2020; Bellavia et al. 2023; Boney et al. 2026).

### Animals

Mature female outbred OF1 mice (*n* = 76), 3–6 months old were housed in an animal facility under controlled temperature (22 °C ± 2 °C) and light (12 h light/day) conditions, with water and food provided ad libitum. The experiments were performed in accordance with the national regulations on the protection of animals used for scientific and educational purposes and conformed to the European Union Council Directive 2010/63/EU of September 22, 2010. They were reported according to the ARRIVE guidelines (https://arriveguidelines.org).

### Oocyte isolation

Mice were sacrificed, ovaries were dissected, placed in M2 culture medium (M7167; Sigma-Aldrich, St. Louis, MO, USA) and punctured with the tips of sharp tweezers to release the oocytes from the antral follicles into the medium. Cumulus cells, if present, were removed from the oocytes by gentle pipetting. Only oocytes at GV stage were selected for further experiments. Depending on the experimental scheme oocytes were maintained at the GV stage for 24 h prior to further processing (Experiment 1, Experiment 3) or immediately placed in culture for meiotic maturation (Experiment 2).

**Fig. 1:**
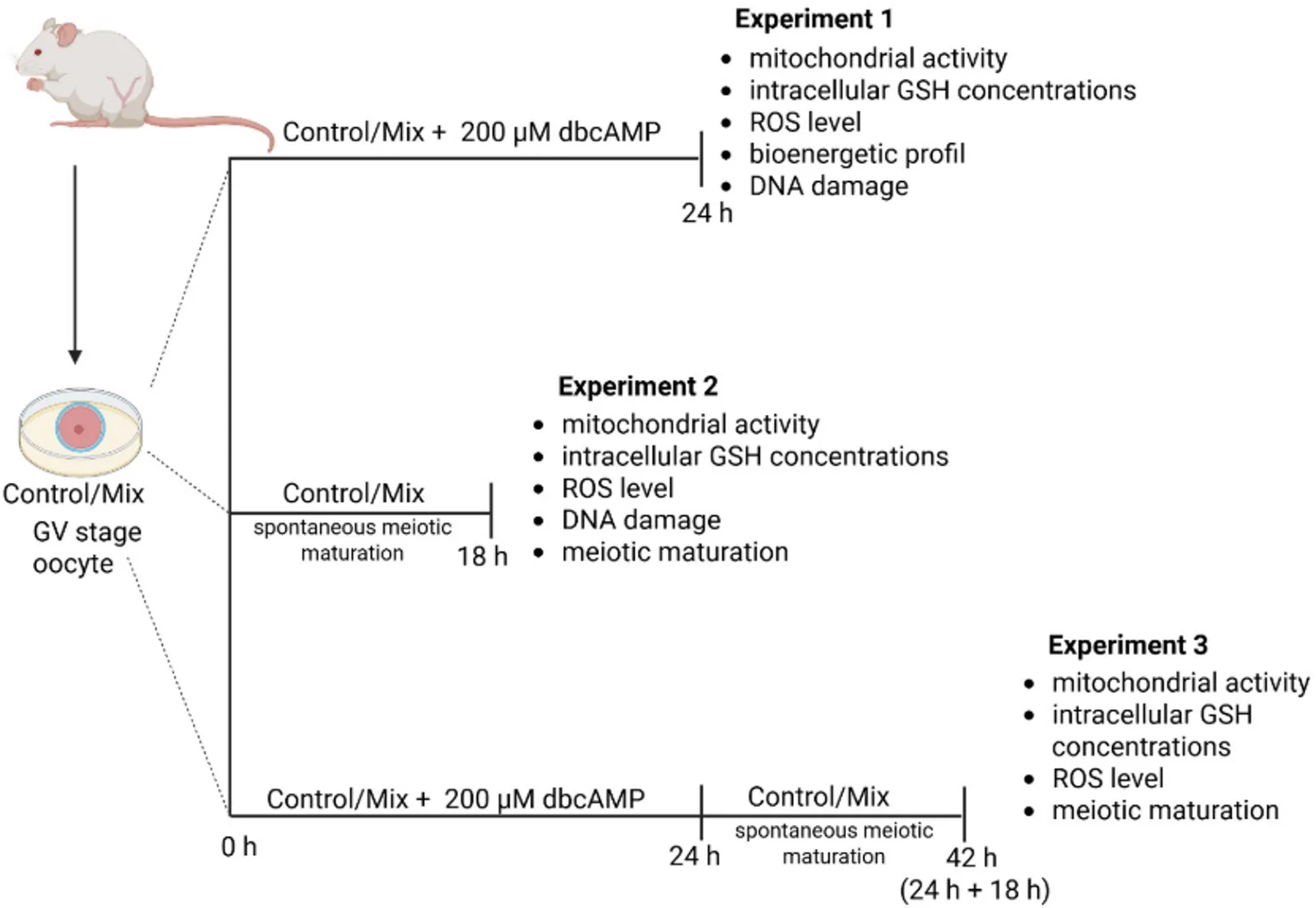
Schematic representation of the experimental design and timeline used to investigate the effects of Mix on mouse oocytes. Graphic created in BioRender. https://BioRender.com/on5e0ab

### Experimental design

*Experiment 1*. Effect of 24-hour PFAS exposure on GV stage oocytes (13–15 per group) (Fig. 1). Oocytes isolated from ovaries were placed in 10 µL drops of M2 medium (control group) or in M2 medium with PFAS mixture (experimental group, Mix), covered with mineral oil (M8410, Sigma-Aldrich, St.Louis, MO, USA). To both media, 200 µM N6,2′-O-Dibutyryladenosine 3′,5′-cyclic monophosphate sodium salt (dbcAMP; D0627, Sigma-Aldrich, St.Louis, MO, USA) was added, to retain oocytes at the GV stage. Oocytes were cultured for 24 h at 37 °C in 5% CO_2_ containing air and next subjected to the measurement of mitochondrial activity, intracellular GSH concentrations, ROS levels, bioenergetic profile, and DNA damage.

*Experiment 2*. Effect of 18-hour PFAS exposure during the oocyte in vitro meiotic maturation (IVM) (Fig. 1). After isolation from the ovaries, the GV stage oocytes (15–16 per group) were placed in 10 µL drops of M2 medium (control group) or M2 medium containing Mix (treated group), covered with mineral oil and cultured at 37 °C in 5% CO_2_ containing air to allow meiotic maturation After 18 h, the efficiency of meiotic maturation was evaluated, and only oocytes which reached MII stage were collected for the measurement of mitochondrial activity, intracellular GSH concentrations, ROS levels, and DNA damage.

*Experiment 3*. Effect of continuous exposure to PFAS during the 24-hour incubation at the GV stage, followed by a further 18-hour exposure during meiotic maturation (Fig. 1). The GV stage oocytes (12–14 per group) were placed in 10 µL drops of M2 medium (control group) or M2 medium containing (experimental group). In both groups oocytes were kept at GV stage by addition to the medium dbcAMP as described above. Petri dishes containing drops with oocytes were covered with mineral oil and left for 24 h at 37 °C in 5% CO_2_ containing air. After 24 h, oocytes were washed to remove dbcAMP (2 × 5 min in M2 medium), placed in fresh M2 medium (control group) or fresh M2 medium containing Mix (treated group) and incubated at 37 °C in a 5% CO_2_ atmosphere or 18 h to allow meiotic maturation. At the end of the 18-h incubation period, the efficiency of meiotic maturation was evaluated and MII stage oocytes were subjected to measurements of mitochondrial activity, intracellular GSH concentrations, and ROS levels.

### Assessment of in vitro meiotic maturation of mouse oocytes

The oocytes were examined after 18 h of in vitro incubation to evaluate the overall efficiency of meiotic maturation. On this basis, the oocytes were placed into three categories: GV oocytes (oocytes that had not resumed meiosis), GVBD oocytes (oocytes that had resumed meiosis by progressing through GVBD but had not extruded the first polar body), and MII oocytes (oocytes that had extruded the first polar body and were arrested at the second meiotic metaphase (MII), thereby completing meiotic maturation).

### Assessment of mitochondrial activity and mitochondrial membrane potential in mouse oocytes

To assess mitochondrial activity the oocytes were labelled with MitoTracker Deep Red at concentration of 500 nM (M22426; Invitrogen, Paisley, UK) and incubated for 30 min at 37 °C. Then, following two washes with M2, images of the oocytes were obtained using an Axiocam 503 bright-field/fluorescence microscope (Zeiss, Jena, Germany) at the excitation and emission wavelengths of 644 and 665 nm, respectively. The fluorescent images were analysed using ImageJ Software (National Institutes of Health, Bethesda, MD, USA). Individual oocytes were manually contoured to calculate the average signal intensity. Signal intensities were measured in four areas adjacent to the oocytes from four different sides and averaged to obtain the average background signal intensity in each image. The average background intensity was subtracted from the average signal intensity of each oocyte analysed in the image. Data are reported relative to the average fluorescence intensity of control oocytes (set to 100) in each experiment.

### Assessment of reactive oxygen species levels and intracellular glutathione concentration in mouse oocytes

ROS levels were assessed using the 10 µM fluorescent probe 2–7′-dichlorofluorescein diacetate (H2DCFDA; D399; Invitrogen, Paisley, UK). Oocytes were incubated in M2 medium containing the dye for 30 min at 37 °C, washed twice in M2 medium, and then imaged using an Axiocam 503 bright-field/fluorescence microscope with excitation wavelengths 495 nm and emission wavelengths 527 nm. To assess GSH concentration, the oocytes were incubated with 50 µM monochlorobimane (mBCl; 69899; Sigma-Aldrich, St.Louis, MO, USA) for 45 min at 37 °C in M2 medium. Then, after two washes with M2 medium, images of the oocytes were obtained using an Axiocam 503 bright-field/fluorescence microscope with excitation wavelengths 380 nm and emission wavelengths 461 nm. The fluorescent images were analysed using ImageJ Software as described above.

### Measurement of nuclear and mitochondrial DNA damage

The number of lesions in nuclear DNA (nDNA) and mitochondrial DNA (mtDNA) of single oocytes was assessed using the LORD-Q (long-run real-time PCR-based DNA damage quantification) method (Lehle et al. 2014; Dannenmann et al. 2017), according to the previously published protocol (Kotarska et al. 2024). Briefly, control and PFAS-exposed MII oocytes were individually lysed in 40 µl of Taq Buffer (B38; Thermo Scientific, Waltham, MA, USA) containing proteinase K (0.2 mg/ml) (1019-20-5; A&A Biotechnology, Gdańsk, Poland) by incubation for 30 min at 55 °C, followed by 10 min at 95 °C. The obtained lysates were used directly as DNA templates in real-time PCR reactions. For nDNA, a long fragment (probe) and an internally nested short fragment (normalization control) of repetitive L1 elements were amplified (L1-LORD-Q). For mtDNA, amplification of long and nested short fragments of a single sequence in mitochondrial genome was performed (classical LORD-Q). The sequences of primers are presented in Table 1. Taq polymerase and standard SYBR Green I dye were used for amplification and real-time detection of the short fragments. To ensure proper amplification of the long fragments, high-fidelity and rapid PrimeSTAR GXL polymerase was employed in combination with ResoLight dye. The reaction mixtures for the short fragments of both genomes contained: 2 µl of DNA template, PowerUp SYBR Green Master Mix (Applied Biosystems, Waltham, MA, USA), and 200 nM of each primer, in a total volume of 10 µl per well. Real-time analysis was performed using the QuantStudio 5 Real-Time PCR System (Applied Biosystems). The cycling conditions were as follows: 2 min at 50 °C, 2 min at 95 °C, followed by 40 cycles of 15 s at 95 °C and 1 min at 60 °C, and subsequent melt curve analysis. The reaction mixtures for the long fragments of both genomes contained: 2 µl of DNA template, 0.4 µl of PrimeSTAR GXL Polymerase (Takara, Kusatsu, Japan), PrimeSTAR GXL Buffer (Takara), dNTP mix (200 µM each) (Takara), primers (200 nM each), and 0.05 µl of ResoLight Dye (Roche, Basel, Switzerland), in a total volume of 10 µl per well. Real-time analysis was carried out on the LightCycler 96 System (Roche). The cycling conditions were as follows: 1 min at 98 °C; 40 cycles of 10 s at 98 °C, 15 s at 60 °C, and 35 s at 68 °C; followed by melt curve analysis. All samples were run in triplicate. In each 96-well PCR plate, serial two-fold dilutions of standards were included to assess reaction efficiency. Mean Ct values for amplification of long and short fragments, together with amplification efficiencies, were used in a comparative ΔΔCt-based formula to calculate the number of DNA lesions per 10 kb (Lehle et al. 2014). The number of lesions in individual oocytes was calculated relative to the control oocyte exhibiting the lowest DNA damage, which was assumed to be damage-free.

**Table 1:** Sequences of primers used in LORD-Q assays (5–3′)

Genome	Product length (bp)	Forward	Reverse
Nuclear	66	GGCGAGGATGTGGAGAAAG	GTGGTTGTACAAGCCTGCA
	3494	CAGCCACAAGAACAGAATGC	
Mitochondrial	78	CGAGAAGAGGGGCATTGGTG	ATCCCCTTCCCCATTTGGTC
	3255	AGCTACCCCCAAGTTTAATGG	TCCTACTGGTCCGATTCCA

### Seahorse analysis

The MitoStress Test kit (Agilent, Santa Clara, USA) was used according to the manufacturer’s instructions using a Seahorse XFp analyzer (Agilent). Prior to the test, the medium was changed to Seahorse XF DMEM test buffer pH 7.4 supplemented with 2 mM glutamine, 1 mM pyruvate and 10 mM glucose. A total of 180 µl of XF assay medium was added to each well, and the oocytes were transferred from the dish to the plate and incubated in a CO_2_ free incubator for 1 h. Oxygen consumption rate (OCR) was measured after the addition of oligomycin (1.5 µM), carbonyl cyanide-4 (trifluoromethoxy) phenylhydrazone (FCCP; 2 µM), and rotenone and antimycin A (Rot/AA, 0.5 µM). Using a combination of oligomycin, an inhibitor of mitochondrial ATP synthase (complex V), FCCP, which is a decoupling agent that collapses the proton gradient and disrupts the mitochondrial membrane potential, and Rot/AA, inhibitors of mitochondrial ETC complexes I and III, key parameters of mitochondrial function were assessed by directly measuring oxygen consumption rate (OCR) in GV-stage oocytes in real time, using an Agilent Seahorse analyzer. Data were analyzed using Seahorse Wave software (Seahorse Bioscience, Santa Clara, USA) and then exported to Prism 8 (GraphPad, La Jolla, CA, USA) for statistical analysis. Data were normalized to the number of oocytes per well.

### Analysis of the spindle assembly checkpoint (SAC) activity

After 3 h of culture for meiotic maturation, oocytes were synchronized by selecting those that had undergone GVBD. Following an additional 4 h of culture (7 h total), the selected oocytes were transferred to the medium supplemented with AZ3146, a potent inhibitor of the SAC pathway (Daszkiewicz et al. 2025). 18 h after the start of culture, the oocytes were classified according to meiotic stage.

### Statistical analysis

All experiments were performed in at least three independent replicates. Normal distribution was analysed by the Shapiro–Wilk test, and homogeneity of variance was tested by Levene’s test. The data were expressed as the mean ± SD. Student’s t-test was used for comparisons of data between two groups conforming to normal distribution and homogeneity of variance, while non-normal distribution data were analysed using the Mann-Whitney U test. *P* < 0.05 indicated statistically significant differences (GraphPad Software, La Jolla, CA, USA).

## Results

### PFAS exposure of GV stage oocytes induces nuclear DNA lesions, alters mitochondrial activity and influences the balance between GSH and ROS levels

**Fig. 2:**
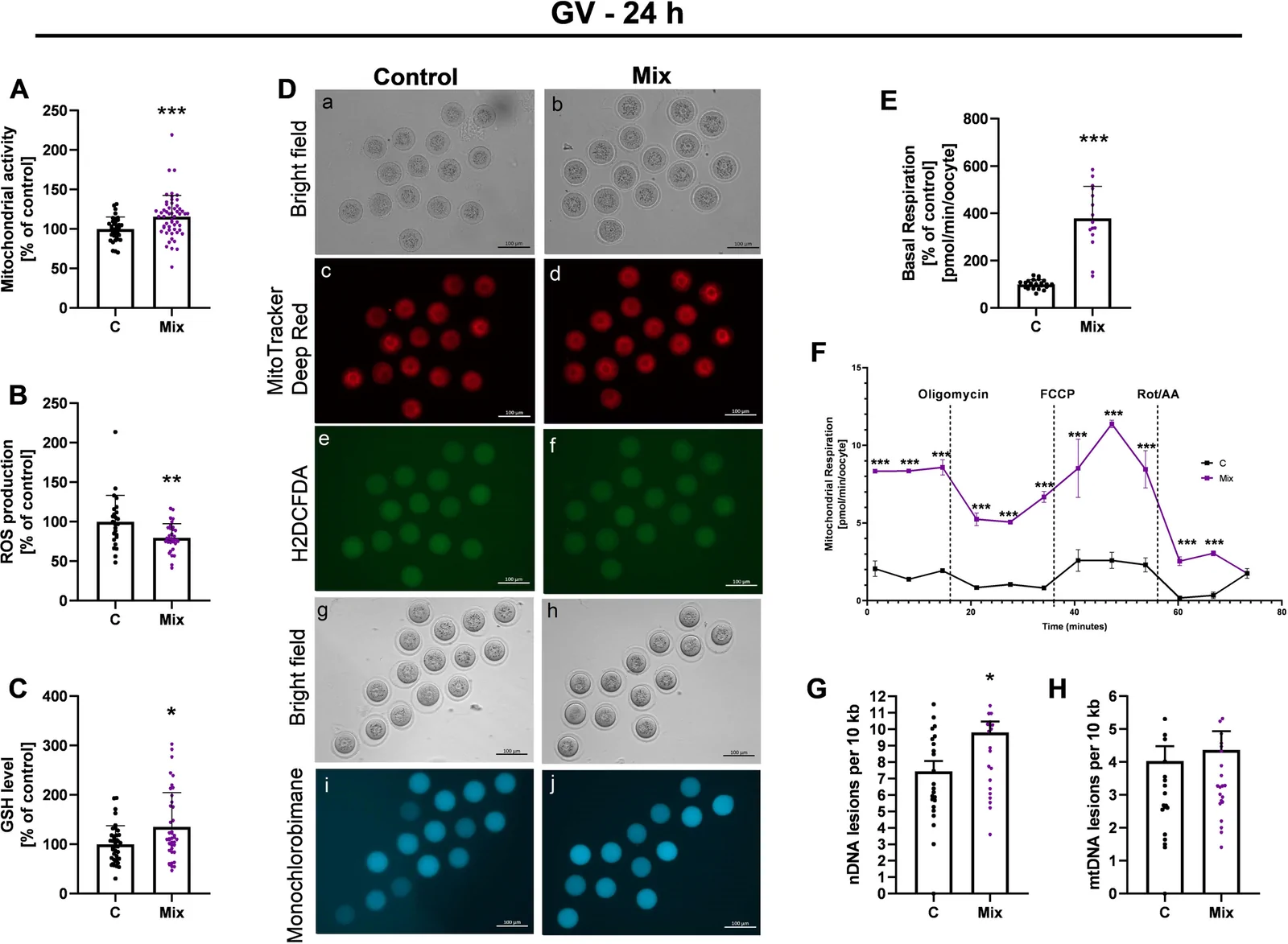
The mitochondrial activity and respiration, antioxidant system, DNA damage in mouse GV stage oocytes in response to 24 h exposure to PFAS mixture. The effect of Mix (PFOS at 224 ng/mL, PFOA at 145 ng/mL, PFHxS at 213 ng/mL, PFDA at 9 ng/mL, PFHpA at 6 ng/mL, PFUnDA at 4 ng/mL, and PFNA at 20 ng/mL) on the mitochondrial activity (**A**), ROS production (**B**) GSH level (**C**), basal OCR (**E**) and the OCR (**F)**, nuclear DNA (**G**) and mitochondrial DNA damage (**H**) in GV stage oocytes after 24 h of exposure. Representative imagines (**D**) of MitoTracker Deep Red staining, indicating mitochondrial activity (**c**, **d**), H2DCFDA labelling, indicating ROS production (**e**, f), and monochlorobimane staining, indicating GSH level (**i**, **j**). Error bars denote the mean ± standard deviations (SD); Each dot on the graph represents a single oocyte, derived from three independent biological replicates. *P < 0.05, **P < 0.01, ***P < 0.001 vs. control (C)

The GV stage is the immature, resting phase of an oocyte in the ovary. Ovaries contain a finite pool of these primary oocytes in resting follicles. Exposure of these oocytes to the PFAS mixture caused a 15% increase in the level of active mitochondria compared with the control group (*p* < 0.001; Fig. 2A). At the same time, a significant elevation in reduced GSH content was observed in the treated oocytes (135 vs. 100% in the control group; *p* < 0.01; Fig. 2C), accompanied by a 21% reduction in reactive oxygen species (ROS) production (*p* < 0.01; Fig. 2B). Measuring mitochondrial function using the Seahorse XF analyzer showed that the oxygen consumption rate (OCR) was significantly increased in the group treated with Mix (*p* < 0.001; Fig. 2E), and the OCR profile over time showed that treated oocytes revealed an increase in maximum respiration (*p* < 0.001; Fig. 2F). Additionally, DNA integrity analysis showed that 24-hour treatment of GV stage oocytes with Mix led to a significant increase in the number of nuclear DNA (nDNA) lesions (*p* < 0.05; Fig. 2G), however, without any significant effect on mitochondrial DNA (mtDNA) (Fig. 2H).

### The PFAS mixture impedes meiotic maturation of mouse oocytes through SAC activation

**Fig. 3:**
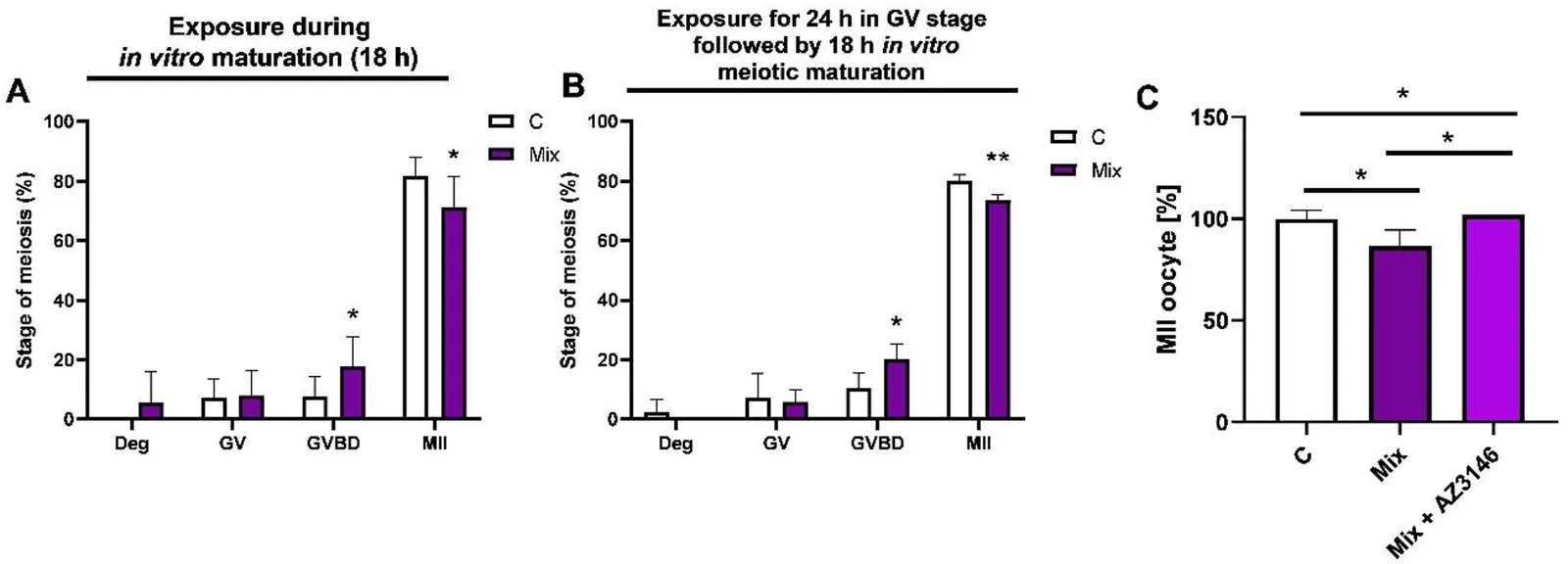
The progression of meiotic maturation in vitro of oocytes exposed to PFAS. The effect of Mix (PFOS at 224 ng/mL, PFOA at 145 ng/mL, PFHxS at 213 ng/mL, PFDA at 9 ng/mL, PFHpA at 6 ng/mL, PFUnDA at 4 ng/mL, and PFNA at 20 ng/mL) on the efficiency of in vitro maturation after 18 h of exposure during meiotic maturation (**A**), after 24 h exposure at GV stage followed by a further 18 h of exposure during meiotic maturation (**B**), and upon simultaneous treatment with PFAS and SAC inhibitor AZ3146 (**C**). Error bars denote the mean ± standard deviations (SD) *P < 0.05, **P < 0.01 vs. control (C). Deg - degenerated, GV - germinal vesicle, GVBD - germinal vesicle breakdown, MII - metaphase II

Next, we analyzed the efficiency of meiotic maturation of oocytes that were exposed to Mix during in vitro maturation for 18 h (Experiment 2), as well as those continuously exposed for 24 h at GV stage followed for a further 18 h exposition during spontaneous in vitro meiotic maturation (Experiment 3). Exposure to a mixture of PFAS disrupted oocyte maturation in both groups by blocking the extrusion of the first polar body (PB1), thus impairing the transition from the germinal vesicle breakdown (GVBD) stage to the metaphase II (MII) stage. In effect we observed a significantly higher proportion of oocytes arrested at GVBD stage (17.72 vs. 7.72%, *p* < 0.05; Fig. 3A), accompanied by a reduced proportion of oocytes reaching the MII stage (71 vs. 81% *p* < 0.05; Fig. 3A) upon PFAS exposure during in vitro maturation. Similarly, exposure for 24 h followed by a further 18 h of exposure during in vitro meiotic maturation led to a significantly higher proportion of oocytes remaining at the GVBD stage (20 vs. 10% in the control, *p* < 0.05; Fig. 3B) and a lower proportion of oocytes reaching the MII stage (73 vs. 80%, *p* < 0.01; Fig. 3B).

The reason for the elevated frequency of GVBD arrest upon PFAS treatment might be the activation of SAC, a cellular mechanism arresting the cell cycle progression at the metaphase when genomic integrity is endangered. To check this possibility, we performed oocyte maturation in the presence of PFAS mixture as well as AZ3146, a potent inhibitor of the SAC pathway. Under these conditions all oocytes extruded PB1 and reached MII stage, compared with 85% of oocytes completing maturation in the presence of PFAS only (*p* < 0.05; Fig. 3B). These results indicate that PFAS exposure activates the SAC which blocks PB1 extrusion by inducing oocyte arrest at MI stage.

### Completion of meiotic maturation in the presence of PFAS mixture results in nDNA damage with no effect in mitochondrial activity and ROS/GSH balance

**Fig. 4:**
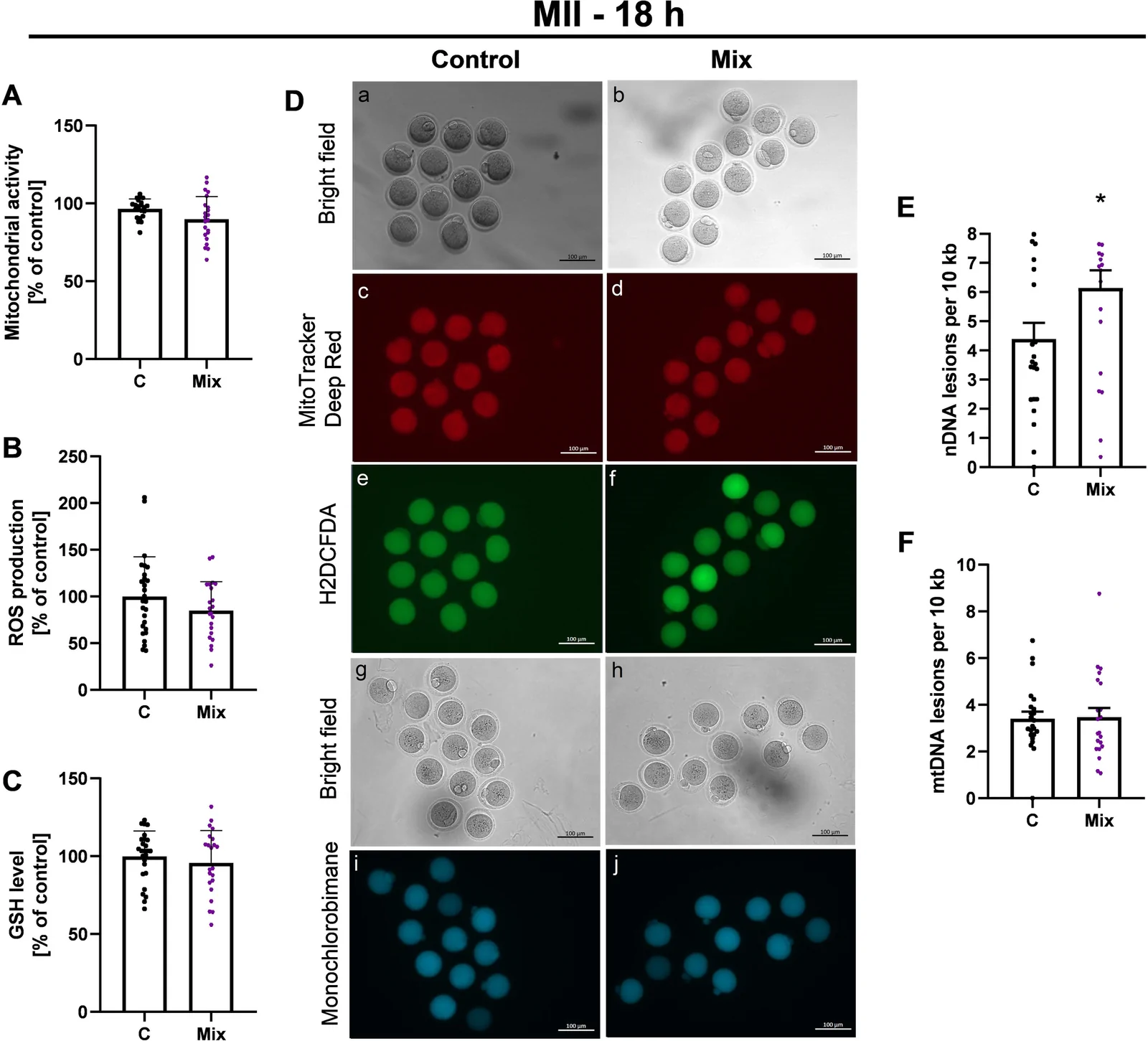
Mitochondrial activity, ROS production as well as GSH and DNA damage levels in mouse MII oocytes undergoing meiotic maturation in the presence of PFAS. The effect of treatment of mouse oocytes with Mix (PFOS at 224 ng/mL, PFOA at 145 ng/mL, PFHxS at 213 ng/mL, PFDA at 9 ng/mL, PFHpA at 6 ng/mL, PFUnDA at 4 ng/mL, and PFNA at 20 ng/mL) on mitochondrial activit (**A**), ROS production (**B**), GSH level (**C**), nuclear DNA (nDNA) (**E**) and mitochondrial DNA (mtDNA) damage (**F**) after 18 h. Representative images (**D**) of MitoTracker Deep Red staining, indicating mitochondrial activity (c, d), H2DCFDA labelling, indicating ROS production (e, f), and monochlorobimane staining, indicating GSH level (i, j). Error bars denote the mean ± standard deviations (SD). Each dot on the graph represents a single oocyte, derived from three independent biological replicates. *P < 0.05 vs. control (C)

A significant fraction of oocytes exposed to the PFAS mixture during meiotic maturation arrested at MI. The majority, however, completed maturation by reaching the MII stage. We decided, therefore, to check the status of the oocytes that which underwent the full maturation to MII in the presence of PFAS. Mitochondrial activity, ROS production, as well as GSH level in these oocytes remained comparable to those in control MII oocytes (Fig. 4A-D). However, the level of nDNA damage increased by 40% compared to the control group (*p* < 0.05; Fig. 4E), while no change in the level of mtDNA damage was observed (Fig. 4F).

### Exposure to the PFAS mixture for 24 h followed by 18 h of in vitro meiotic maturation does not affect mitochondrial activity or ROS and GSH levels in MII mouse oocytes

**Fig. 5:**
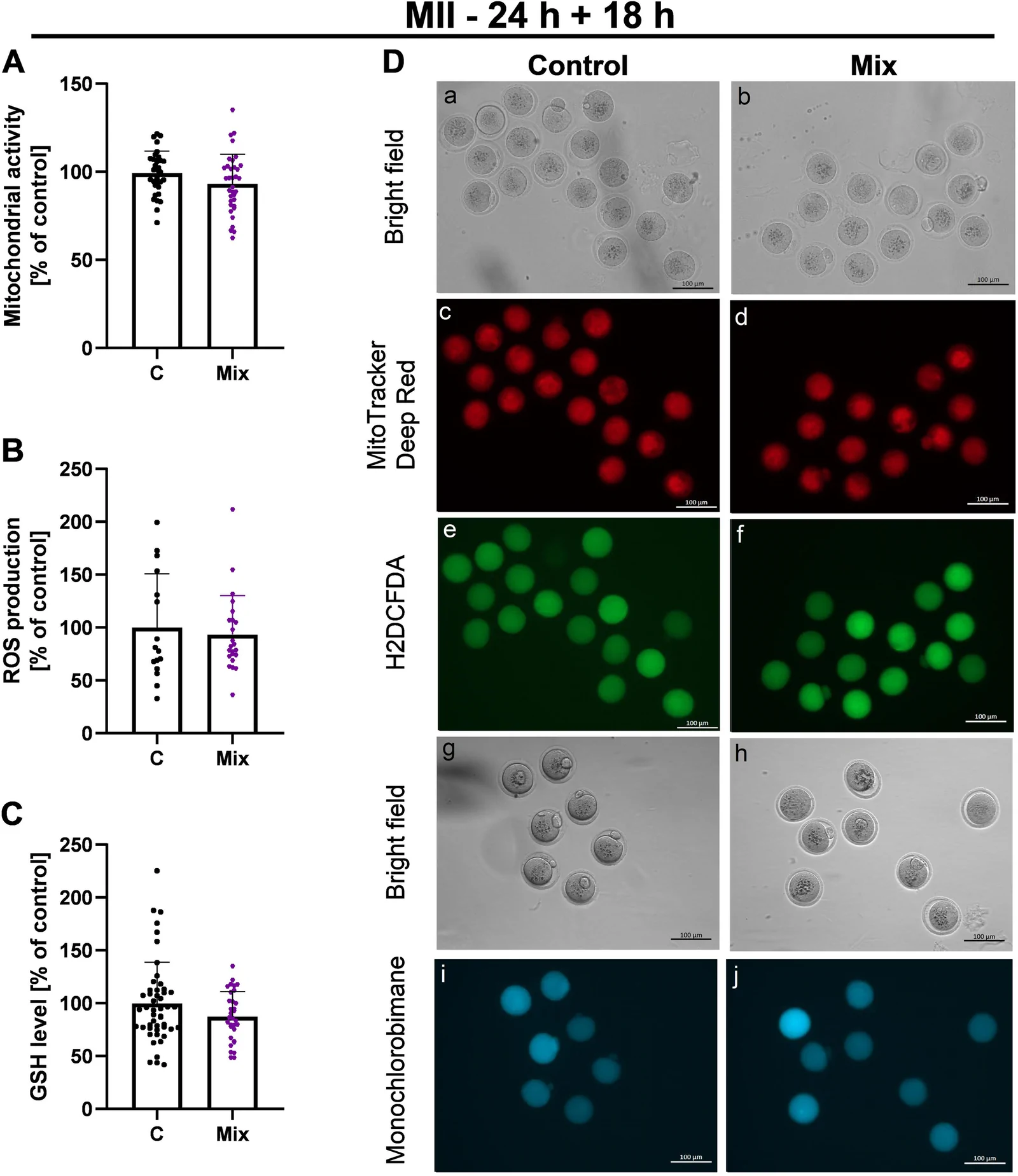
The effects of a 42-hour exposure to a PFAS mixture on mitochondrial activity, and antioxidant system in mouse MII stage oocytes. The effect of treatment of mouse oocyte with Mix (PFOS at 224 ng/mL, PFOA at 145 ng/mL, PFHxS at 213 ng/mL, PFDA at 9 ng/mL, PFHpA at 6 ng/mL, PFUnDA at 4 ng/mL, and PFNA at 20 ng/mL) on mitochondrial activity (**A**), ROS production (**B**) and GSH level (**C**) after 24 h + 18 h. Representative imagines (**D**) of MitoTracker Deep Red staining, indicating mitochondrial activity (c, d), H2DCFDA labelling, indicating ROS production (e, f), and monochlorobimane staining, indicating GSH level (i, j). Error bars denote the mean ± standard deviations (SD). Each dot on the graph represents a single oocyte, derived from three independent biological replicates

Since no changes in mitochondrial activity or GSH and ROS levels were observed in MII oocytes exposed to the PFAS mixture for 18 h during in vitro maturation, an attempt was made to assess the effect of prolonged exposure (24-h exposure in GV stage followed by a further 18-h exposure during in vitro maturation). Despite the extended exposure period, no statistically significant changes in mitochondrial activity or intracellular GSH and ROS levels were found (Fig. 5A-C).

## Discussion

Oocytes are extremely sensitive cells, whose quality and developmental competence depend on a precise metabolic balance, redox balance, and genome integrity (Yuan et al. 2012; Li et al. 2020; Richani et al. 2021). Any factor that disrupts these processes can lead to impaired oocyte maturation, fertilization, and embryonic development. Therefore, the overall aim of this study was to determine the effect of a mixture of PFAS, corresponding in composition and concentration to compounds detected in human follicular fluid (FF) (Kim et al. 2020; Bellavia et al. 2023; Boney et al. 2026) on oocyte meiotic maturation, their mitochondrial function, and DNA integrity. Our results show that PFAS mixture imposes an arrest at MI during meiosis through activation of the SAC, thereby reducing the number of oocytes completing maturation. Furthermore, exposure to PFAS at the GV stage as well as during meiotic maturation causes nDNA damage. Interestingly, we have observed elevated PFAS-induced mitochondrial activity, which disrupts the balance between GSH and ROS, but only in oocytes at the GV stage.

In recent years, the potential impact of PFAS on female reproductive cells has received increasing attention due to the chemicals’ stability and presence in body fluids, such as follicular fluid (Kim et al. 2020; Bian et al. 2025). Previous studies reported deleterious effect of PFAS on meiotic maturation, ROS level, mitochondrial function, and DNA integrity in mammalian oocytes (Chen et al. 2021; Jiao et al. 2021; Zhang et al. 2022; Feng et al. 2023). However, these compounds were tested individually or in relatively high doses. Meanwhile, PFAS exhibit complex and context-dependent effects, including non-monotonic dose–response relationships, where low and high concentrations may elicit distinct cellular responses. The biological activity of PFAS may also differ when compounds are tested individually compared to mixtures, due to additive, synergistic, or antagonistic interactions. This partly unpredictable nature of these substances was addressed in a very recent study demonstrating that simultaneous in vivo exposure to PFOS and PFOA, both at doses closer to physiologically relevant levels, induces a spectrum of effects in oocytes including those observed in our study, such as meiotic arrest, changes in ROS level and mitochondrial activity, as well as DNA damage (Yan et al. 2025). The key strength of the present study, therefore, is the use of very complex PFAS mixture at doses and ratios of the compounds that reflect those measured in human FF (Kim et al. 2020), thereby providing physiologically relevant assessment of PFAS-related risks to female reproductive competence.

It is known that the progression through meiotic maturation is guarded by the SAC which monitors the correct and stable attachment of chromosomes to microtubules (Lara-Gonzalez et al. 2012). Once the attachments are established, the SAC is silenced, enabling the oocyte to extrude the PB1 and complete maturation by reaching MII. We have found that a significant fraction of PFAS-exposed oocytes that underwent GVBD did not extrude PB1, however simultaneous inhibition of the SAC pathway restored completion of meiotic maturation. This proves that PFAS-induced inhibition of PB1 extrusion is caused by activation of the SAC which arrests oocytes at MI. In mammalian oocytes, DNA damage triggers the SAC to induce MI arrest (Collins et al. 2015; Marangos et al. 2015), thereby reducing the risk of abnormal embryo formation. Consistently, we have found that the oocytes that matured in the presence of PFAS exhibited an elevated frequency of DNA lesions. This strongly suggests that in the group of oocytes which underwent PFAS-induced arrest in meiotic progression at MI, the level of DNA damage exceeded the threshold level for SAC activation. To our knowledge, there are no previous data documenting the effect of PFAS on SAC activation. However, in line with our findings, previous studies have shown that PFNA (Jiao et al. 2021), and PFOS with PFOA (Feng et al., 2023) hamper progression through meiotic maturation in mouse oocytes by blocking the MI to MII transition. Based on our results, SAC activation appears to be a protective response, as PFAS exposure induces nDNA damage already at the GV stage. However, the persistence of elevated DNA damage in MII oocytes raises serious concern regarding the developmental competence of these oocytes that, in spite of PFAS exposure, escape SAC arrest and proceed through meiosis.

Interestingly, we observed that PFAS elevate mitochondrial activity and oxygen consumption rate in GV-stage oocytes, an effect which, to our knowledge, has not been reported before. Mitochondria are a major source of ROS, generating them as by-products of oxidative metabolism. Glutathione (GSH), on the other hand, acting as a primary antioxidant that neutralizes ROS to prevent cellular damage, maintains redox balance, and supports proper maturation, fertilization, and early embryo development (Hansen et al. 2015; Ren et al. 2025). We observed that PFAS increased GSH level and reduced ROS production despite maintaining higher mitochondrial activity in oocytes at GV- but not at MII-stage. This observation suggests that immature GV-stage oocytes are more sensitive to PFAS exposure, which can disrupt the delicate balance between mitochondrial activity, ROS production, and GSH levels. Mammalian oocytes remain physiologically blocked at the GV stage from the fetal period until ovulation—for months in mice and even for decades in humans. Given the ability of PFAS to cross the placental barrier and reach the fetus (Alteri et al. 2025), this implies a potential long-term impact of these compounds on oocytes at one of the most sensitive stages of oogenesis.

Importantly, our data reveal a stage-specific vulnerability of oocytes to PFAS exposure. Immature GV-stage oocytes exhibit increased mitochondrial activity and oxygen consumption, accompanied by altered redox homeostasis, characterized by elevated GSH levels and reduced ROS. This adaptive response suggests an attempt to counteract PFAS-induced metabolic stress, yet it simultaneously highlights the fragility of the mitochondrial–redox balance at this early stage. An issue emerges to what extent (and at what cost) oocytes exposed to PFAS pollution under natural conditions can continuously engage such counteracting mechanism throughout the prolonged physiological arrest at GV stage. In contrast, MII oocytes appear less responsive in terms of mitochondrial and redox changes, despite harboring significant DNA damage, underscoring a potentially silent risk to embryo quality and fertility.

In summary, we have demonstrated for the first time that a mixture of PFAS at concentrations and compositions relevant to human exposure disrupts oocyte maturation, genome integrity, and mitochondrial function. By identifying SAC activation and stage-dependent metabolic disturbances as key targets of PFAS toxicity, our study advances the understanding of how persistent environmental pollutants may contribute to female subfertility and adverse reproductive outcomes.

### Research data deposit

Research data will be deposited in Krakow Open Research Data Repository (RODBUK) upon publication.

## References

[CR1] Alteri A, Canosa S, Di Nisio A, Foresta C, Pisaturo V (2025) The silent crisis: investigating the impact of environmental pollutants on embryo-fetal development: a narrative review of the Group of Special Interest for Environment of the Italian Society of Fertility and Sterility and Reproductive Medicine. J Assist Reprod Genet 42(12):4103–4114 41032203 10.1007/s10815-025-03653-9 PMC12705484

[CR29] Bellavia A, Zou R, Bjӧrvang RD, Roos K, Sjunnesson Y, Hallberg I, Holte J, Pikki A, Lenters V, Portengen L, Koekkoek J, Lamoree M, Van Duursen M, Vermeulen R, Salumets A, Velthut-Meikas A, Damdimopoulou P (2023) Associationbetween chemical mixtures and female fertility in women undergoing assisted reproduction in Sweden and Estonia. Environ Res 216:1114447. 10.1016/j.envres.2022.114447 PMC9729501 36181890

[CR3] Bian Y, Yue Y, Cheng Y, Wang D, He L, Yan P, Song H, Wang T, Zhou W, Zhang X, Pan Z, Liu L (2025) Associations of per- and polyfluoroalkyl substances in follicular fluid with polycystic ovarian syndrome in infertile women may be mediated by sex hormones. Front Public Health 13:1526910 10.3389/fpubh.2025.1526918 PMC12185472 40556922

[CR4] Boney SS, Christen C, Wetmore BA, Murr AS, Raburn D, Young SL, Abraham C, Stoker TE (2026) First evidence of legacy and emerging per- and polyfluoroalkyl substances (PFAS) in the follicular fluid of a cohort of North Carolina in vitro fertilization (IVF) patients. Reprod Toxicol 139:109102. 10.1016/j.reprotox.2025.109102 41197966 PMC13487813

[CR5] Chen J, Miao Y, Gao Q, Cui Z, Xiong B (2021) Exposure to perfluorooctane sulfonate in vitro perturbs the quality of porcine oocytes via induction of apoptosis. Environ Pollut 284:117508. 10.1016/j.envpol.2021.117508 34261219

[CR6] Collins JK, Lane SIR, Merriman JA, Jones KT (2015) DNA damage induces a meiotic arrest in mouse oocytes mediated by the spindle assembly checkpoint. Nat Commun 6:8553. 10.1038/ncomms9553 26522232 PMC4659839

[CR7] Dannenmann LB, Lorscheid S, Huber S, Essmann SM, Schulze-Osthoff FK (2017) Simultaneous quantification of DNA damage and mitochondrial copy number by long-run DNA-damage quantification (LORD-Q). Oncotarget 8:112417–112411. 10.18632/oncotarget.20112 29348835 PMC5762520

[CR8] Daszkiewicz, Gąsior R, Kotarska Ł, Polański KZ (2025) Oocyte checkpoint response to the spindle poison depends on the cytoplasm volume. Syst Biology Reproductive Med. 10.1080/19396368.2025.2576084 41144805

[CR9] Ding N, Harlow SD, Batterman S, Mukherjee B, Park SK (2020) Longitudinal trends in perfluoroalkyl and polyfluoroalkyl substances among multiethnic midlife women from 1999 to 2011: the Study of Women’s Health Across the Nation. Environ Int 135:105381 31841808 10.1016/j.envint.2019.105381 PMC7374929

[CR10] Feng J, Soto-Moreno EJ, Prakash A, Balboula AZ, Qiao H (2023) Adverse PFAS effects on mouse oocyte in vitro maturation are associated with carbon-chain length and inclusion of a sulfonate group. Cell Prolif 56(2):e13353. 10.1111/cpr.13353 36305033 PMC9890540

[CR11] Hallberg I, Persson S, Olovsson M, Moberg M, Ranefall P, Laskowski D, Damdimopoulou P, Marc-Andre S, Ruegg J, Sjunnesson YCB (2022) Bovine oocyte exposure to perfluorohexane sulfonate (PFHxS) induces phenotypic, transcriptomic, and DNA methylation changes in resulting embryos in vitro. Reprod Toxicol 109:19–30. 10.1016/j.reprotox.2022.02.004 35219833

[CR12] Hansen JM, Harris C (2015) Glutathione during embryonic development. Biochimica et Biophysica Acta (BBA) -. Gen Subj 1850(8):1527–1542. 10.1016/j.bbagen.2014.12.001 25526700

[CR13] Jiao X, Liu N, Xu Y, Qiao H (2021) Perfluorononanoic acid impedes mouse oocyte maturation by inducing mitochondrial dysfunction and oxidative stress. Reprod Toxicol 104:58–67. 10.1016/j.reprotox.2021.07.002 34246765 PMC8477654

[CR14] Kim YR, White N, Bräunig J, Vijayasarathy S, Mueller JF, Knox CL, Harden FA, Pacella R, Toms LL (2020) Per- and poly-fluoroalkyl substances (PFASs) in follicular fluid from women experiencing infertility in Australia. Environ Res 190:109963. 10.1016/j.envres.2020.109963 32745751

[CR15] Kotarska K, Gąsior Ł, Rudnicka J, Polański Z, Lane S, Kauppi L (2024) Long-run real-time PCR analysis of repetitive nuclear elements as a novel tool for DNA damage quantification in single cells: an approach validated on mouse oocytes and fibroblasts. Anim GeneticsCellular Mol Life Sci 65:181–190. 10.1007/s13353-023-00817-0 PMC10789673 38110826

[CR16] Lara-Gonzalez P, Westhorpe FG, Taylor SS (2012) The spindle assembly checkpoint. Curr Biol 22:R966–R980. 10.1016/j.cub.2012.10.006 23174302

[CR17] Lehle S, Hildebrand DG, Merz B, Malak PN, Becker MS, Schmezer P, Essmann F, Schulze-Osthoff K, Rothfuss O (2014) LORD-Q: a long-run real-time PCR-based DNA-damage quantification method for nuclear and mitochondrial genome analysis. Nucleic Acids Res 42:e41. 10.1093/nar/gkt1349 24371283 PMC3973301

[CR18] Li R, Luo Y, Xu J, Sun Y, Ma Z, Chen S (2020) Effects of oxygen concentrations on developmental competence and transcriptomic profile of yak oocytes. Zygote 6: 459–469. 10.1017/S0967199420000337 32772955

[CR19] Marangos P, Stevense M, Niaka K, Lagoudaki M, Nabti I, Jessberger R, Carroll J (2015) DNA damage-induced metaphase I arrest is mediated by the spindle assembly checkpoint and maternal age. Nature communications 2;6:8706. 10.1038/ncomms9706 PMC4667640 26522734

[CR20] Polański Z (2013) Spindle assembly checkpoint regulation of chromosome segregation in mammalian oocytes. Reprod Fertility Dev 25:472–483. 10.1071/RD12145 22951024

[CR21] Ren J, Liu G, Cui H, Dong X, Mao S, Liu Y, Dai Y (2025) Effect of glutathione addition on vitrification of ovine oocytes. Theriogenology 240:117412. 10.1016/j.theriogenology.2025.117412 40179567

[CR22] Richani D, Dunning KR, Thompson JG, Gilchrist RB (2021) Metabolic co-dependence of the oocyte and cumulus cells: essential role in determining oocyte developmental competence. Hum Reprod Update 27(1):27–47. 10.1093/humupd/dmaa043 33020823

[CR23] Wang Y, Schatten H, Cui XS, Sun SC (2021) Quality Control of Mammalian Oocyte Meiotic Maturation: Causes, Molecular Mechanisms and Solutions. Fronties Cell Dev Biology 9:736331. 10.3389/fcell.2021.736331 PMC8440897 34540849

[CR24] Yan XZ, Peng J, Liu YQ, Fan RN, Ni XY, Gong L, Zhang DN, Huang X, Tan SH, Wang HL (2025) Mixed exposure to PFOA and PFOS induces oocyte apoptosis and subfertility in mice by activating the Hippo signaling pathway. Reprod Toxicol 132:108829. 10.1016/j.reprotox.2024.108829 39746460

[CR25] Yao X, Liu W, Xie Y, Xi M, Xiao L (2023) Fertility loss: negative effects of environmental toxicants on oogenesis. Front Physiol 14:1219045. 10.3389/fphys.2023.1219045 37601637 PMC10436557

[CR26] Yuan Y, Wheeler MB, Krisher RL (2012) Disrupted redox homeostasis and aberrant redox gene expression in porcine oocytes contribute to decreased developmental competence. Biol Reprod 87(4):78. 10.1095/biolreprod.112.099952 22811572

[CR27] Zhang P, Qi C, Ma Z, Wang Y, Zhang L, Hou X (2022) Perfluorooctanoic acid exposure in vivo perturbs mitochondrial metabolic during oocyte maturation. Environ Toxicol. 10.1002/tox.23652 36029293

[CR2] Zou A, Bjӧrvang R, Roos RD, Sjunnesson K, Hallberg Y, Holte I, Pikki J, Lenters A, Portengen V, Koekkoek L, Lamoree J, Van Duursen M, Vermeulen M, Salumets R, Velthut-Meikas A, Damdimopoulou AP (2023) Association between chemical mixtures and female fertility in women undergoing assisted reproduction in Sweden and Estonia. Environ Res 216:1114447. 10.1016/j.envres.2022.114447 PMC9729501 36181890

